# Supplementary material for: Ragulator and GATOR1 complexes promote fission yeast growth by attenuating TOR complex 1 through Rag GTPases
Source: eLife. 2017 Dec 4;6:e30880. doi: 10.7554/eLife.30880 (PMC5752196; doi:10.7554/eLife.30880)
Supplement: Figure 5—source data 1. — Sequence alignment of the N-terminal regions of the RagA/B orthologs (UniProtKB IDs: Hs RagA, Q7L523; Hs RagB, Q5VZM2-1; Dm RagA, Q9VHJ4; Sc Gtr1, Q00582; Sp Gtr1, O74824) and that of K-Ras (P01116-1) by the CLUSTALW program (http://www.genome.jp/tools/clustalw/) is shown. Among the Ras-superfamily GTPases, the boxed Ser/Thr residues are conserved and their substitutions have been used to generate RagA/B and K-Ras mutants predicted to be restricted to the GDP-bound conformations in a number of studies including those listed in the table below. Gtr1 Ser20 mutated in the study by Ma et al. (2016) is marked by a red square. [file elife-30880-fig5-data1.docx]

| GDP-locked mutants of RagA/B | Mutation site | Organism | References |
| --- | --- | --- | --- |
| RagA T21N | Thr-21 | *H. sapiens* | [1−4] |
| RagB T54N | Thr-54 | *H. sapiens* | [1, 4−9] |
| RagA T16N | Thr-16 | *D. melanogaster* | [1] |
| Gtr1 S20N | Ser-20 | *S. cerevisiae* | [10, 11] |
| Gtr1 S16N | Ser-16 | *S. pombe* | This study |


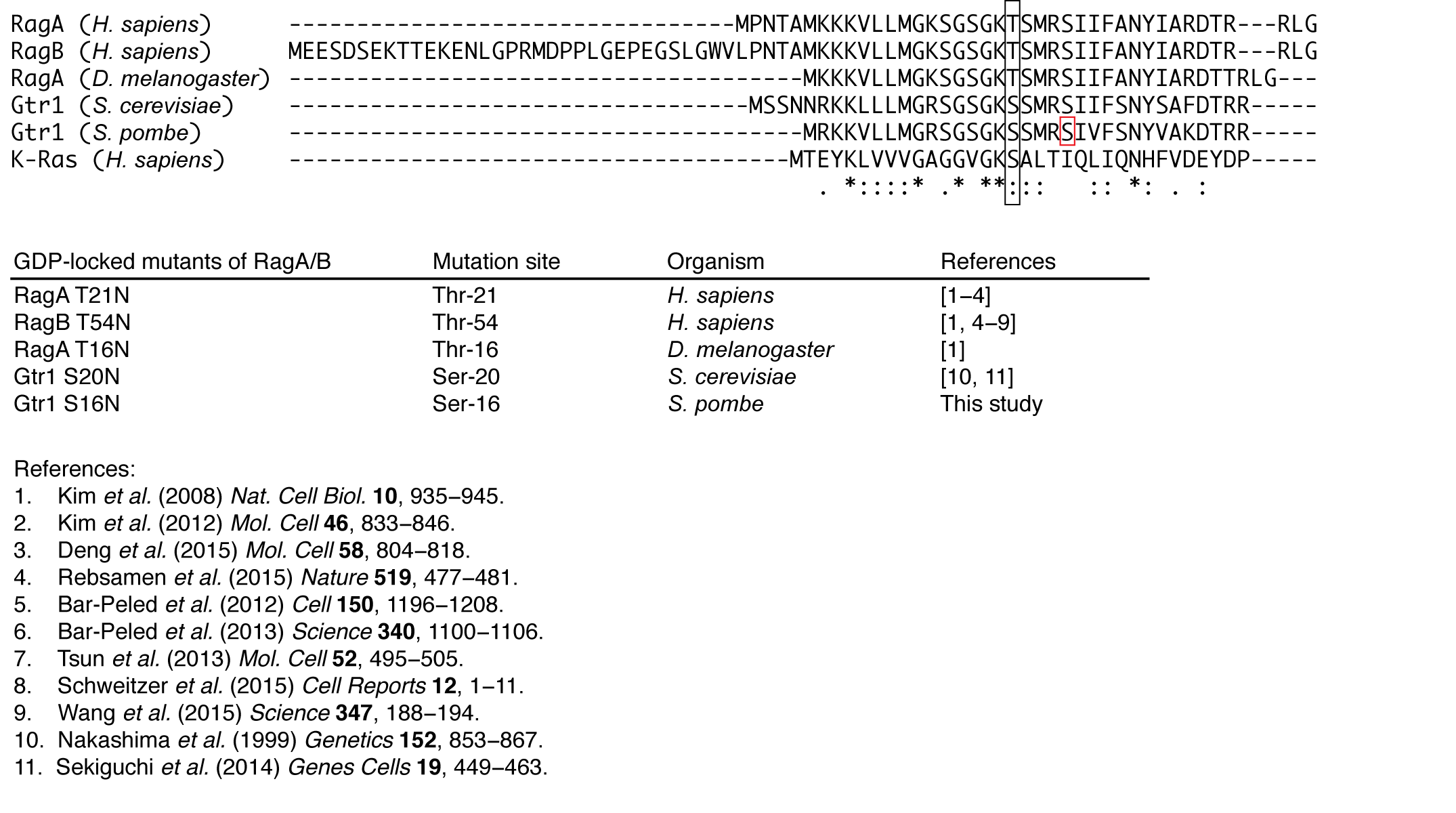


References:

1. Kim *et al.* (2008) *Nat. Cell Biol.* **10**, 935−945.
2. Kim *et al.* (2012) *Mol. Cell* **46**, 833−846.
3. Deng *et al.* (2015) *Mol. Cell* **58**, 804−818.
4. Rebsamen *et al.* (2015) *Nature* **519**, 477−481.
5. Bar-Peled *et al.* (2012) *Cell* **150**, 1196−1208.
6. Bar-Peled *et al.* (2013) *Science* **340**, 1100−1106.
7. Tsun *et al.* (2013) *Mol. Cell* **52**, 495−505.
8. Schweitzer *et al.* (2015) *Cell Reports* **12**, 1−11.
9. Wang *et al.* (2015) *Science* **347**, 188−194.
10. Nakashima *et al.* (1999) *Genetics* **152**, 853−867.
11. Sekiguchi *et al.* (2014) *Genes Cells* **19**, 449−463.

**Figure 5-source data 1.** Mutation sites used to generate the GDP-locked mutant forms of RagA/B GTPases.

Sequence alignment of the N-terminal regions of the RagA/B orthologs (UniProtKB IDs: *Hs* RagA, Q7L523; *Hs* RagB, Q5VZM2-1; *Dm* RagA, Q9VHJ4; *Sc* Gtr1, Q00582; *Sp* Gtr1, O74824) and that of K-Ras (P01116-1) by the CLUSTALW program (http://www.genome.jp/tools/clustalw/) is shown. Among the Ras-superfamily GTPases, the boxed Ser/Thr residues are conserved and their substitutions have been used to generate RagA/B and K-Ras mutants predicted to be restricted to the GDP-bound conformations in a number of studies including those listed in the table below. Gtr1 Ser20 mutated in the study by Ma *et al*. (2016) is marked by a red square.
